# Inraoperative and Histological Visualization of Disrupted Vulnerable Plaques following Diagnostic Angiography of Moderate Carotid Stenosis

**DOI:** 10.4061/2010/602642

**Published:** 2010-01-17

**Authors:** Tatsushi Mutoh, Tatsuya Ishikawa, Akifumi Suzuki, Nobuyuki Yasui

**Affiliations:** ^1^Department of Stroke Science, Research Institute for Brain and Blood Vessels-Akita, Akita 010-0874, Japan; ^2^Department of Surgical Neurology, Research Institute for Brain and Blood Vessels-Akita, Akita 010-0874, Japan

## Abstract

*Background*. Digital subtraction angiography (DSA) remains an important tool for diagnosis of carotid stenosis but is associated with risk for periprocedural complications. This is the first report of direct intraoperative and histolopathologic visualization of DSA-related carotid plaque disruption. *Case*. A 64-year-old man diagnosed to have a 60% right carotid stenosis received diagnostic DSA for therapeutic decision-making. He developed transient left hand numbness and weakness immediately after the procedure. Intraoperative imaging during carotid endarterectomy revealed a fragile plaque with sharp surface laceration and intraplaque hemorrhage at the bifurcation. Microscopy of the specimen demonstrated a large atheromatous plaque with fibrous hypertrophy and intraplaque hemorrhage filled with recent hemorrhagic debris. *Conclusion*. The visualized carotid lesion was more serious than expected, warning the danger of embolization or occlusion associated with the catheter maneuvers. Thus the highest level of practitioner training and technical expertise that ensures precise assessment of plaque characteristics should be encouraged.

## 1. Introduction

Despite recent advances in minimally invasive imaging techniques for carotid vessels like Doppler ultrasonography (US), MR angiography (MRA), and CT angiography (CTA), digital subtraction angiography (DSA) provides the highest spatial resolution and still remains the “gold standard” for diagnosis of carotid artery stenosis, but it is associated with risk for procedure-related neurologic complications. In fact, therapeutic decisions in large clinical trials [1–4] have been based on maximal internal carotid artery (ICA) stenosis depicted with conventional DSA.

It is well known that vulnerable carotid plaque is an atheromatous plaque that is particularly prone to disruption, fracture, or fissuring with a higher risk for embolization, occlusion, and consequent ischemic neurological events [5]. Although disruption of such unstable plaques has been commonly implicated as a risk for procedure-related neurological complications in patients undergoing DSA, most resultant stroke events are clinically silent or transient [6, 7], and there are few descriptions of the affected vessel walls.

We report a case of direct visualization and histolopathologic examination of carotid plaque disruption associated with the diagnostic DSA for therapeutic consideration of asymptomatic moderate-grade carotid stenosis, which was incidentally detected later during carotid endarterectomy (CEA).

## 2. Case Report

**Figure 1:**
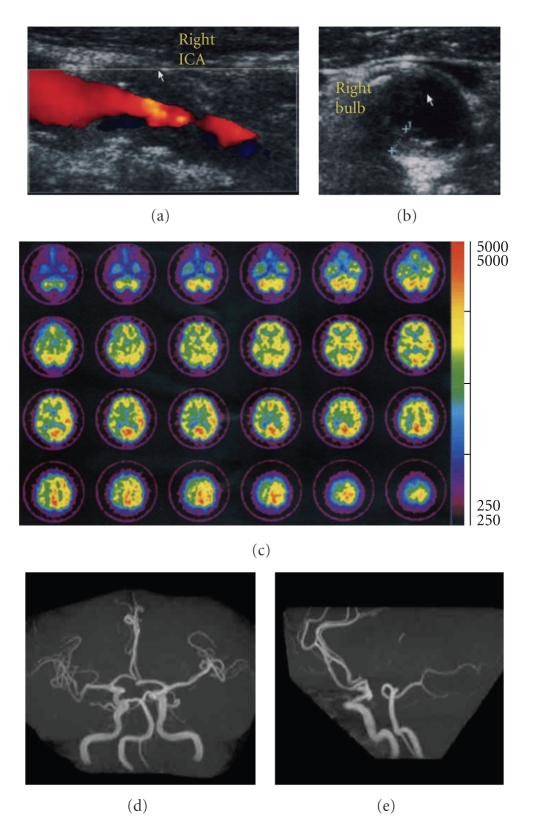
(a) B-mode ultrasound with color flow Doppler image on the longitudinal display of the carotid plaque with irregular surface. (b) The transverse display of the plaque at the carotid bifurcation. (c) ^123^I-IMP SPECT transaxial slices of a patient with right ICA stenosis. MR angiography ((d) submental vertical projection; (e) lateral projection) with a hypoplastic or absent anterior and posterior communicating arteries.

A 64-year-old man with past medical history of hypertension, type 2 diabetes mellitus, hypercholesterolemia, and symptomatic ischemic stroke in the territory of the thalamoperforate artery diagnosed to have an asymptomatic moderate (approximately 50%) right ICA stenosis that was observed on MRA was referred to our center for consideration of surgical intervention. Carotid Doppler ultrasonography demonstrated hypoechoic plaques with an irregular surface at the carotid bifurcation extending to the proximal ICA with stenosis of 83% (by area method) and peak systolic flow velocity at 1.89 m/s (Figures 1(a) and 1(b)). Resting single photon emission CT (SPECT) showed severe hypoperfusion in the right ICA territory (Figure 1(c)), presumably due to less prevalence of collateral flow via the anterior or posterior communicating artery (Figures 1(d) and 1(e)) [8].

**Figure 2:**
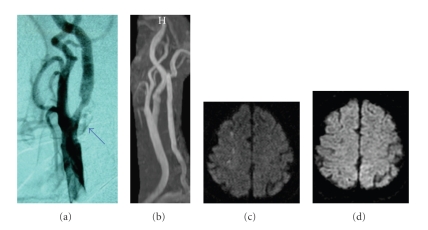
(a) Peroperative common carotid angiogram (lateral projection) of a 64-year-old asymptomatic patient with a 60% stenosis of the right ICA by NASCET criteria (*arrow*). (b) Postoperative MR angiography confirmed disappearance of the stenosis. (c) Axial diffusion-weighted MR imaging of the brain immediately after the occurrence of neurologic events following the DSA. Ipsilateral hyperintense lesions are appreciable at the cortical-subcortical junction of right front-parietal lobes. (d) Postoperative diffusion-weighted imaging indicative of no ischemic lesions associated with CEA. Postoperative MR imagings ((b) and (d)) were performed on the next day after CEA.

For therapeutic decision-making, diagnostic carotid angiography was then performed via a femoral approach. It was difficult to cannulate a 5-Fr. JB-2 catheter (Cook, Bloomington, IN) over an angled 0.035 inch Radifocus guidewire (Terumo, Tokyo, Japan) selectively advanced into the right common carotid artery (CCA). The procedure was repeated with a 5-Fr. Simmons II catheter (Cook, Bloomington, IN) but failed to engage in the CCA due to severe vascular elongation. Therefore, the guidewire was advanced carefully, with special attention not to cross the stenotic lesion at the proximal ICA, into the lingual branch of the external carotid artery (ECA) for support, and the catheter was successfully advanced to the CCA. The DSA revealed a 60% stenosis of the proximal right ICA with wall irregularities (Figure 2(a)), calculated according to the North American Symptomatic Carotid Endarterectomy Trial (NASCET) method [2]. The contralateral carotid angiogram demonstrated a mild stenosis in the posterior wall of the ICA. Vertebral angiography was discontinued as it was difficult to probe the bilateral vessels due to elongation.

The patient developed numbness and mild weakness of the left hand immediately after the procedure. Diffusion-weighted MR imaging showed multiple, small hyperintense lesions in the distal ICA territory of the right front-parietal lobe indicative of an embolic origin from the carotid plaques (Figure 2(c)). The symptoms were transient and resolved within 24 hours of the procedure with supplemental intravenous fluids followed by oral clopidogrel (Plavix, Sanofi Pharmaceuticals, New York, NY) 75 mg once daily. Based on the results of the Asymptomatic Carotid Atherosclerosis Study (ACAS) [1] and the Medical Council Asymptomatic Carotid Surgery Trial (ACST) [4], the patient is considered to be a good candidate for elective surgery and given informed consent about CEA.

**Figure 3:**
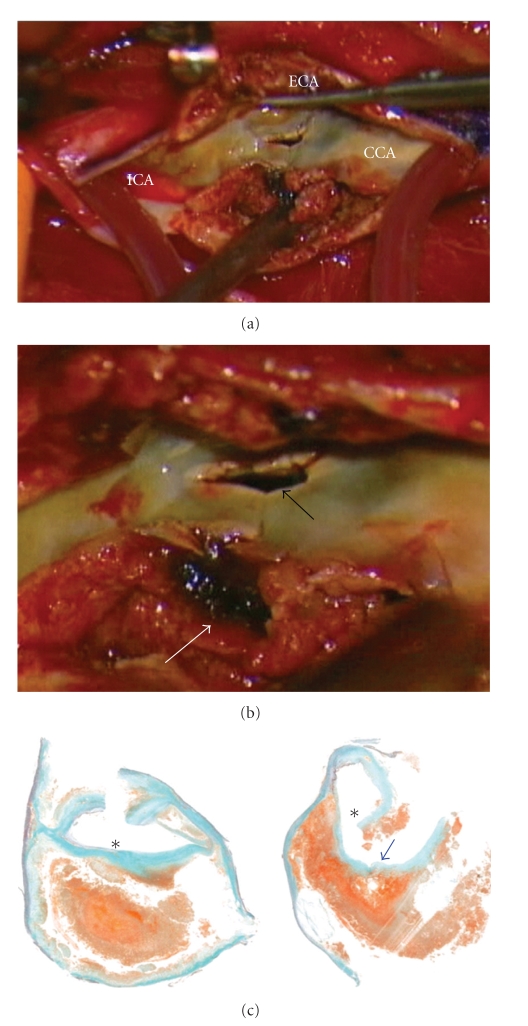
(a) An intraoperative view of an atheromatous plaque originating from the proximal ICA during CEA with a shunt. (b) Magnified image revealing the sharp laceration of the plaque surface (*black arrow*) and intraplaque hemorrhage (*white arrow*). (c) Elastica-Masson stain matching histology cross section of the carotid plaque showing a large lateralized atheroma and intraplaque hemorrhage. A ditch shown in *blue arrow* suggests a part of the lacerating injury. Asterisks indicate the lumen.

Two weeks later, the patient underwent successful right CEA. Fragile atherosclerotic plaque with sharp surface laceration, somewhat different from atheromatous plaque rupture, accompanied by intraplaque hemorrhage was observed at the proximal ICA close to the bifurcation (Figures 3(a) and 3(b)). There was no evidence of perforation outside the wall. Microscopic examination of the endarterectomy specimen revealed a large atheromatous plaque with fibrous hypertrophy and intraplaque hemorrhage filled with recent hemorrhagic debris that stained red to brown with Elastica-Masson stain, cholesterol crystal formation, and speckled calcification (Figure 3(c)). The postoperative course was uneventful and the stenosis had improved significantly on follow-up MRA (Figure 2(b)) with no apparent distal embolization (Figure  2(d)). The patient was asymptomatic at his neurological baseline without any postoperative complications and was discharged on postoperative day 10.

## 3. Discussion

Although minimally invasive MRA and CTA have partially replaced conventional DSA in clinical routine, it is still not a standardized method for detection and grading of carotid artery stenosis, especially for asymptomatic patients with moderate carotid stenosis (50%–69%) where the quantification of stenosis can seriously impact on clinical decision-making.

According to the current United States guidelines from the American Academy of Neurology, surgical treatment of asymptomatic patients with carotid stenosis 60%–99% in patients with a 5-year life expectancy are recommended if the operator has a perioperative complication rate of <3% (level A) [9]. By contrast, there is no treatment recommendation for asymptomatic patients with stenosis 50%–59%. This patient has already diagnosed as 50% moderate stenosis on MRA, and thus confirmatory imaging was required for therapeutic decision-making. Although multidetector CT had initially been taken into consideration for assessment of carotid stenosis; however, conventional DSA was chosen after all because we were worried about misclassification of patients within the surgical range associated with greater underestimation with CTA in moderate grades of stenosis [10].

It should be noted that the high diagnostic accuracy of DSA before deciding on carotid intervention must be balanced against the risk of neurological complications. The neurologic complications are more common when indication for DSA is carotid stenosis or ischemic stroke (1.8%) [11]. Furthermore, it has also been pointed out that such clinically overt neurological symptoms are only the “tip of the iceberg” since the rates of DSA-related silent microemboli detected by diffusion-weighted MR abnormalities are considerably higher [7, 12, 13]. Although it remains a matter of debate, the level of operator experience (procedural and fluoroscopy time, multiple catheters use, and aortography) and the nature of the underlying disease are thought to be predictors of the occurrence of cerebral ischemic events following diagnostic DSA [7, 11]. At the authors' institution, diagnostic cerebral angiographies are generally performed by neurosurgical fellows who had already performed at least 250 cerebral angiographies and allowed to perform the procedures on their own, with an acceptable neurologic complication rate (0.8%) compatible to those of recent data [7, 11, 14].

We postulate that the periprocedural manipulation could have resulted in the symptomatic neurological event. This might be related to the difficulties in probing the vessels, the presence of vulnerable atherosclerotic plaque located at the carotid bifurcation that might be impinged and scraped off the vessel wall presumably during the guidewire maneuver in the ECA, and the instability of fresh thrombus in exulcerating plaques that might embolize during the procedure. Unfortunately, the surface morphology of the excised carotid plaque corresponding to the affected intimal lesion could not be well characterized in this case because of artifacts introduced during the removal of the lesion, and nor could we distinguish the hemorrhagic lesion caused directly by the angiographical procedure and/or by its natural course of bleeding. During CEA, the full thickness of the plaque was incised, thereby disrupting the luminal surface of the lesion. However, the disrupted plaque showed sharp laceration (Figure 2(b)) that cannot be explained simply by atheromatous plaque rupture.

## 4. Conclusion

This is, to the authors' knowledge, the first report of direct visualization and histolopathologic examination of DSA-related carotid plaque disruption. The impact of the invasive diagnostic carotid angiography on vessel wall injuries warns the danger of serious procedure-related complications, and thus the highest level of practitioner training and technical expertise that ensures precise preprocedural assessment of plaque characteristics by multimodal methods (e.g., ulcerations, surface irregularities, number of plaques, echolucent plaques, plaque distribution along the carotid bifurcation, intraplaque hemorrhage, and lipid-rich necrotic core) [5, 15, 16] should be encouraged in the patient subgroup where the DSA remains necessary.
